# Behavioral Interventions to Prevent or Delay Dementia: Protocol for a Randomized Comparative Effectiveness Study

**DOI:** 10.2196/resprot.8103

**Published:** 2017-11-27

**Authors:** Glenn Smith, Melanie Chandler, Dona EC Locke, Julie Fields, Vaishali Phatak, Julia Crook, Sherrie Hanna, Angela Lunde, Miranda Morris, Michelle Graff-Radford, Christine A Hughes, Susan Lepore, Andrea Cuc, Maria Caselli, Duane Hurst, Jennifer Wethe, Andrea Francone, Jeanne Eilertsen, Pauline Lucas, Charlene Hoffman Snyder, LeeAnn Kuang, Marigrace Becker, Pamela Dean, Nancy Diehl, Marvin Lofquist, Shirley Vanderhook, Diana Myles, Denise Cochran

**Affiliations:** ^1^ Department of Clinical and Health Psychology University of Florida Gainesville, FL United States; ^2^ Department of Psychiatry and Psychology Mayo Clinic Florida Jacksonville, FL United States; ^3^ Department of Psychiatry and Psychology Mayo Clinic Arizona Scottsdale, AZ United States; ^4^ Department of Psychiatry and Psychology Mayo Clinic Minnesota Rochester, MN United States; ^5^ Department of Neurological Sciences University of Nebraska Medical Center Omaha, NE United States; ^6^ Department of Health Sciences Research Mayo Clinic Florida Jacksonville, FL United States; ^7^ University of Washington Seattle, WA United States; ^8^ VA Puget Sound Seattle, WA United States; ^9^ Patient and Partner Advisory Group Minneapolis, MN United States; ^10^ Patient and Partner Advisory Group Davis, CA United States

**Keywords:** cognition disorders, dementia, secondary prevention, behavioral research

## Abstract

**Background:**

Currently, people at risk for dementia and their caregivers are confronted with confusing choices about what behavioral interventions are most effective.

**Objective:**

The objective of this study is to determine which empirically supported behavioral interventions most impact the outcomes highly valued by patients with mild cognitive impairment and their partners.

**Methods:**

This protocol describes a comparative effectiveness trial targeting 300 participants with mild cognitive impairment and their study partners. The trial is being conducted at the Mayo Clinic campuses in Arizona, Florida, Minnesota, and the University of Washington in Seattle. The study examines the contribution of five behavioral interventions (yoga, memory compensation training, computerized cognitive training, support groups, and wellness education) on primary outcomes of participant and partner quality of life and self-efficacy. In this unique 10-day multicomponent intervention, groups of couples were randomized to have one of the five interventions withheld while receiving the other four. Although the longitudinal follow-up is still under way, enrollment results are available and reported.

**Results:**

In total, 272 couples have been enrolled in the trial and follow-up visits continue. Outcomes will be assessed at the end-of-intervention and 6-, 12-, and 18-month follow-ups. We anticipate reporting on our primary and secondary outcomes across time points in the next 2 years.

**Conclusions:**

This paper describes the protocol for a randomized comparative effectiveness study of behavioral interventions to prevent or delay dementia. We describe of the rationale, design, power analysis, and analysis plan. Also because enrollment is complete and we are in follow-up phases of the study, we have included enrollment data from the trial.

**Trial Registration:**

ClinicalTrials.gov NCT02265757; http://clinicaltrials.gov/ctsshow/ NCT02265757 (Archived by WebCite at http://www.webcitation.org/6ueRfwSYv)

## Introduction

### Scope of the Problem

Alzheimer disease (AD) is the most common cause of dementia. An estimated 5.5 million Americans had AD in 2016, including 454,000 people receiving a new diagnosis of AD every year [1,2]. Roughly 16% of women and 11% of men aged 71 and older have AD. Unless dementia risk can be reduced, it is estimated that by 2050, the number of Americans with AD will triple, exceeding 13 million [1]. Worldwide projections are, for the number of people with AD, to rise from 26.6 million in 2006 to 107 million in 2050 [3]. The cognitive, emotional, behavioral, and functional impact of AD and related dementias is devastating. The majority of people with dementia (75%) live at home where they receive 83% of their care from informal caregivers—unpaid individuals such as family members, friends, and neighbors [1] **.** These caregivers provide valuable services, often at great economic, health, and psychological cost to themselves. Hurd et al [4] estimate the average annual cost of dementia care to be US $56,290, of which more than half is in unpaid care. In 2016, over 18 billion hours of unpaid care were estimated to have been provided by roughly 15.9 million caregivers, translating to nearly US $230 billion in a single year. The stress of caregiving is reflected in 8% higher costs for caregivers’ health compared with noncaregivers [1]. In the later stages, nursing home placement is almost inevitable [5], and those not already qualifying for Medicaid will pay an average of US $81,000 per year for such care [6].

### Early Detection Engenders Secondary Prevention

Dementia prevention through early intervention of risk is now possible. For example, AD has a decade-long predementia phase (ie, a prodrome) that includes reliably identifiable epochs preceding the manifestation of the full syndrome of dementia [7,8]. This understanding has led to new consensus diagnosis criteria for AD that recognizes a prodromal period called mild cognitive impairment (MCI) due to AD [9]. Approximately 15% to 20% of people aged 65 or older have MCI, and it is estimated that 32% to 38% of individuals with MCI develop Alzheimer dementia in 5 years [10]. This concept of MCI fits neatly within a public health framework [11] that identifies three forms of prevention: (1) primary prevention involving interventions to the entire population to reduce risk, (2) secondary prevention involving interventions targeting those at higher risk for a condition, and (3) tertiary prevention involving interventions given to those with disease to mitigate morbidity, such as providing cardiac rehabilitation to reduce the functional impact of heart attack. Nearly all primary prevention models for dementia involve behavioral interventions that improve overall health. For example, it was recently noted that a combined 25% reduction in midlife diabetes, obesity, hypertension, and physical and cognitive inactivity could potentially alleviate 500,000 cases of dementia [12]. Importantly, the emergence of the concept and diagnosis of MCI as a risk state for dementia creates the opportunity for secondary prevention models aimed at delaying (or ideally preventing) progression to dementia. The same interventions proposed for primary prevention show promise as secondary prevention interventions [13]. Moreover, behavioral interventions are growing in popularity and demand by patients with MCI.

Unfortunately, clinical trials with medications for MCI have been uniformly disappointing [14]. However, behavioral interventions involving physical [15] and mental [16] exercise, social engagement [17], and compensation strategies [18,19] have consistently shown positive effects in efforts to delay or prevent dementia. Greater use of behavioral strategies can lessen medication, health care, and long-term care utilization [20]. It is not clear whether these interventions impact the biology of the dementia, but they appear to at least mitigate the functional symptoms it produces, given the above findings. To date, these studies have largely looked at single behavioral interventions. However, dementia is a disorder where multicomponent therapies may be essential to treatment [21].

### A Multi-Modal Behavioral Intervention Program

At Mayo Clinic, we have developed a multicomponent behavioral intervention for patients diagnosed with MCI and a loved one partner called HABIT (Healthy Actions to Benefit Independence and Thinking). This intervention began with a cognitive rehabilitation component because of Mayo Clinic’s long history in cognitive rehabilitation for acquired brain injury. Our study first involved modification of existing compensatory memory techniques and development of a curriculum to help patients with MCI develop procedural memory around using an external memory compensation device. After involvement in brain fitness trials by some of our principal investigators, we added cognitive exercise to the program. We had long offered stand-alone support groups to our patients and partners and developed a partner education series offered in our Alzheimer’s Disease Research Center, and these offerings were eventually also folded into the program that became HABIT. Finally, we opted to add yoga to the programming for its benefit on balance and flexibility improvement and because of literature supporting the benefit of mindfulness practice (which is a feature of yoga) on emotional health and the ease of application of the yoga techniques to individuals across a range of ability levels. Thus, over the period of approximately 3 to 4 years, beginning with cognitive rehabilitation work, HABIT evolved to include 5 components because of the individual work supporting each piece.

This study focuses on the interventions used in the HABIT program. HABIT is a 5-hour-per-day, 5-day-per-week, 2-week program representing an intensive state-of-the-art behavioral and lifestyle approach to preventing progression in MCI. The 5 components are 1 hour each of (1) daily physical exercise, (2) computer-based cognitive exercise (brain fitness), (3) patient and family education, (4) separate support groups for MCI patients and partners, and (5) memory support system compensation training. As described below, there is research to support each of these separate interventions. However, there is little in the way of comparative effectiveness research pitting each component against the other in terms of the highest value for patients and their families.

#### Physical Activity

Exercise interventions are being explored as a means to minimize cognitive decline in MCI. One study observed significant improvement in memory in participants who engaged in a 24-week physical exercise regimen [15]. Participants were encouraged to perform at least 150 min of moderate intensity exercise per week. Walking was most frequently recommended, but participants were free to choose other forms of exercise. However, these participants did not necessarily meet contemporary criteria for MCI.

#### Computer-Based Cognitive Exercise (“Brain Fitness”)

In appropriately designed studies, various investigators, including the principal investigator (PI) of this study, have shown that computerized brain fitness training can improve cognitive function in normal older adults [16,22,23]. Barnes et al [24] reported no significant benefit from brain fitness training in MCI. However, the memory effect size they observed was nearly the same as that reported as significant in the larger study in cognitively normal older adults. This suggests that the Barnes and colleagues’ study (and other studies) may be underpowered to see the modest beneficial effect of computer training in MCI. Other recent trials of a computer training program have suggested modest benefit on cognition and mood in patients with MCI [25]. Belleville et al [26] also reported positive outcomes for a program combining education, computer-based attention training, and internal memory compensatory training. They reported improved list-recall memory and face-name association performance, as well as improvements in subjective memory report and sense of subjective well-being. Another randomized case-control trial of a computer training program demonstrated improvements in story recall, abstract reasoning, and behavioral problems in participants with MCI [27].

#### Education

There is evidence that psychoeducation can bring about positive change in care partners of those with MCI [28,29]. Although most research focuses on dementia education for caregivers, a recent study in patients with MCI found that care partners were more depressed when they had less knowledge of MCI at the time of diagnosis [30]. Studies further show that greater knowledge increases feelings of competence, confidence, and less overall distress in care partners [29].

#### Support Groups

It is becoming apparent that social support is an important aspect of behavioral interventions. For example, research shows that patients with MCI who participate in group therapy learn acceptance of their diagnoses and what lies ahead of them, and care partners become more aware and accepting of cognitive and behavioral problems [31]. A high level of social engagement can reduce the mortality risk in individuals with MCI [32]. More recent studies are reporting similar findings—there is greater acceptance in participants and care partners undergoing group therapy versus wait-list controls [17,33]. Furthermore, a meta-analysis [33] revealed that a higher risk of AD was associated with loneliness and lower levels of social networking and physical activity.

#### Memory Support System Compensation Training

The Memory Support System (MSS) involves use of a portable calendar and note-taking system that includes three sections: (1) appointments, (2) “to-do” items, and (3) a notes section. MSS trainers provide persons with MCI and a care partner training sessions following a structured curriculum of orientation, modeling, practice, and homework assignments. Standardized forms are used daily to document adherence to the process by the trainers as well as to note individuals’ progress. The MCI state is the ideal target for this intervention as people demonstrating MCI typically have “cognitive reserve” in the form of preserved procedural memory even in the face of clinically significant declarative memory impairments [34]. We have specifically studied this strategy and found significant effect sizes on memory-based everyday functions at training end (*d*=1.0), 2-month follow-up (*d*=.88), and 6-month follow-up (*d*=.56) compared with no-treatment controls [18].

As detailed above, multiple kinds of behavioral interventions have shown promise for different outcomes (eg, quality of life [QoL], functioning, cognition, and self-efficacy). Thus, in the HABIT program and in this trial, we opted to measure all of these outcomes, given that individual studies suggest different interventions may impact different outcomes.

Older adults and especially those with cognitive impairment have unique challenges in adhering to behavioral interventions [35,36]. Yet, in our preliminary studies of various combinations of these behavioral interventions, we have found reasonable enrollment, adherence, and excellent retention of patients with MCI and their partners in this type of research [37]. Preliminary comparative effectiveness data comparing MSS with computerized training showed that the MSS is superior to computerized training on a memory-related functional outcome [38], and both MSS and computer training appeared to contribute to improved partner mood in comparison with providing no treatment [39]. The study described herein used the recruitment, intervention, and evaluation infrastructure of the clinical HABIT program to expeditiously determine which behavioral intervention outcomes related to the prevention and delay of dementia are most important to persons with MCI and their partners.

### Aims

Engage patients and care partners who have previously completed a multicomponent behavioral intervention for MCI in the prioritization of outcomes for persons diagnosed with MCI. This aim is complete and under review elsewhere.

Incorporate the results of aim 1 into a study comparing the effectiveness of each of the 5 components of the program with the other components, where the results of aim 1 provide the primary outcome for aim 2.

Demonstrate the use of a novel research design and data analysis method for the evaluation of multicomponent interventions that allows all participants to receive 80% of the intervention.

In aim 1 of this study, we surveyed patients with MCI and their partners who had previously completed the clinical HABIT program. We found these patients and partners ranked QoL and self-efficacy as the most important outcomes for the behavioral interventions to target [40].

In aim 2, we will simultaneously compare all 5 components of the full HABIT program and their respective contributions with these patient-centered outcomes. This program capitalizes on an accomplished multidisciplinary team that included neuropsychologists, dementia educators, exercise specialists, nurse practitioners, social workers, and biostatisticians integrally, as well as substantial existing infrastructure for delivering a multicomponent behavioral intervention developed during prior collaborative grant, “A Multicenter Rehabilitation Intervention for Amnestic Mild Cognitive Impairment” (R01NR012419). That program helped develop resources across the sites involved in this study, which included Mayo Clinic campuses in Minnesota, Arizona, Florida, and the University of Washington.

## Methods

### Participants

Participants in the comparative effectiveness trial were identified from referrals to the HABIT program that come from the neuropsychology and behavioral neurology clinics and/or Alzheimer’s Disease Research Center clinical cores of each participating site. Medical history, symptom profile, physical exam, and neuropsychological testing were reviewed by the neuropsychologists. All subjects had a clinical diagnosis of amnestic MCI (either single domain or multidomain). MCI diagnosis was based on National Institute on Aging-Alzheimer Association criteria [9]. Consecutive HABIT candidates with diagnoses of amnestic MCI (single domain or multidomain) who also met our inclusion/exclusion criteria were approached for consent. The inclusion and exclusion criteria are presented in Textbox 1.

These proposed inclusion and exclusion criteria were vetted and approved by our patient and partner advisory group before the initiation of the study.

Our target was to enroll 300 participants by having each of the 4 sites enroll 75 participants in a 16-month period (5 HABIT sessions with 15 participants each at 4 sites). On the basis of our earlier research, we anticipated only a 10% attrition rate, leaving 270 complete datasets.

#### Randomization

##### Randomization by Subtraction not Addition

At the encouragement of patient and partner advisors, we undertook a nontraditional study design. Traditional randomized controlled trials (RCTs) can be thought of as “additive” trials where randomization leads to the addition of treatments beyond placebo. Participants are confronted with a significant probability of receiving placebo (no treatment). This leads many potential participants to reject participation or to withdraw if they believe they are receiving no treatment. In contrast, this proposed trial was approached as “subtractive.” People were randomized to conditions involving the withholding of one of the five interventions but receiving the other four. This innovative approach to randomization involved suppression of just one of the five treatment components. Thus, all participants received at least 80% of the menu of interventions offered in this trial. Data analysis will focus on determination of which groups had the weakest outcomes as a result of missing a given intervention (see Data Analysis below).

**Inclusion and exclusion criteria.**

Inclusion criteria

- Clinical dementia rating (CDR) scale score ≤0.5
- A cognitively normal care partner screened with the Mini Mental State Exam (MMSE; >24) who has at least twice-weekly contact with the participant
- Either not taking or stable on nootropics for at least 3 months
- Fluent in English (expanding the program to communities of Spanish speakers will be a high priority in subsequent dissemination studies)

Exclusion criteria

- Inclusion in another clinical trial that would exclude participation; subject will be considered for participation at the end of such a trial or as appropriate
- Medically unable to participate in all arms by virtue of visual or auditory impairments or nonambulatory status

##### Randomization of Sessions Not People

In the HABIT program, individualized randomization posed significant risk for diffusion of treatment effects, as the group nature of HABIT permits participants to compare their experiences. There was no blinding of intervention. Thus, we randomized by session. Sessions were offered 4 times per year at each of 4 sites. Thus, in 16 months, we had the opportunity to randomize 20 HABIT sessions (5 each at the 4 sites). We employed block randomization, seeking to assure that randomization to each of the 5 arms of the study resulted in at least 60 participants per arm, and that all sites ran each arm once. All randomization was overseen by Dr Crook (the statistician) and handled by the data management center at Mayo Jacksonville. To avoid bias in enrollment, investigators were not made aware of which arm would be delivered until each session was filled. Participants were not made aware of which arm would be delivered until day 1 of their session.

### Interventions

Our intervention, using the infrastructure of the HABIT program, consisted of 10 days of intervention over 2 weeks. Although the participants were given the weekend off, they were given “homework” to practice each trained component on their own. With one of the five components randomly suppressed in the existing design, each participant and care partner received 4 hours (4 components by 1 hour each) of intervention daily. As noted above, HABIT programming initiates new healthy behavioral habits that MCI patients sustain with the support of care partners cuing, which we implemented within this study.

#### Yoga

Participants engaged in daily 45 to 60 min of yoga. We used yoga as it is suited to the constraints of space and the different levels of baseline physical activity of our participants and partners. We used the HABIT framework, which uses an adapted Hatha Yoga practice where participants sit on chairs for some asanas (poses) and use the chair for support for balance during other standing poses and for others parts of the sequence. This adapted Hatha Yoga style is appropriate for older adults and is both beneficial and accessible for those who have limited mobility, including those with walkers or those who are in wheelchairs. HABIT yoga also incorporates breathing and meditation and cultivates an overall sense of connection and support.

Our instructors had at least 200 hours of training and were certified. The appropriately sequenced HABIT yoga practice met the American College of Sports Medicine recommendation for older adults for muscle strengthening and flexibility. The sessions used an armless sturdy chair placed on top of a sticky mat. Instructions were mirrored for the participants (the instructor faced the students and performed a posture on the left side while instructing the right side to reduce confusion). Breathing practice focused on increasing lung capacity and oxygenation. The sessions included meditation practice to support internal focus.

The HABIT yoga intervention was intended to initiate and sustain a schedule rather than a type of physical activity. Following the 10-day program, participants and partners were encouraged to maintain a schedule of 150 min of their preferred exercise per week. Post program, we consider yoga, swimming, walking, running, or formal exercise programming (water aerobics, resistance training, etc) to count equivalently toward this total. Because most clinical trials of yoga include group classes supported by home practice, we provided a customized DVD (digital video disc) as a supplement for continued use and practice after the program to those who opt to continue yoga. The DVD included sections on the following: poses, modifications, benefits, breathing, and meditation practices.

#### Computerized Brain Fitness Training

We used the commercially available Posit Science product BrainHQ [41] on tablets (eg, iPads). At the time of the study, this product was the latest generation of the BrainFitness auditory processing speed program studied by Smith et al [16] and Zelinski et al [23] (and included components of the Insight visual processing speed program). Participants completed 45 to 60 min of training daily in the program and were encouraged to maintain 150 min of computerized brain training per week for 18 months post program. Participants were provided a 1-year subscription to the program. Each participant’s adherence and progress were tracked through the clinician portal provided by Posit Science, both during HABIT and for 12 months post program.

#### Wellness Education

This education program involved daily 45- to 60-min group lecture sessions with topics including the following: Introduction to the Program, Living with MCI, Changes in Roles and Relationships, Sleep Hygiene, Steps to Healthy Brain Aging, Preventing Dementia, MCI and Depression, Nutrition and Exercise, Assistive Technologies, Participating in Research, and Community Resources.

#### Support Groups

We conducted separate support groups with patients and partners. Group size was limited to 10 members at a maximum. The patient group was focused on reminiscence and adaptation, while the partner groups focused on building resources for coping.

##### Patient

The patient support group met for 45 to 60 min daily. Homework assignments were given in the *LifeBio Memory Journal* and used as a basis for reminiscence-focused group sessions the next day. Patients also accomplished emotional processing around MCI diagnosis and lifestyle impact with a goal toward acceptance and healthy dialog with partners.

##### Partner

The care partner support group met separately from the patient group for 45 to 60 min daily. It involved a traditional support group with no set curriculum, but the following common caregiving themes variously emerged and were addressed in these sessions including the following: Ambiguity of the Diagnosis, Denial, Disclosure to Friends and Family, Role Changes, Communication, Emotional Adjustment, Behavior Changes in Our Loved One, Safety, Driving Issues, Planning for the Future, Caregiver Health, Manufacturing Success, Dementia and Relationships, Communication Skills, Defense Mechanisms, Dimensions of Wellness, Effects on Emotions, Family Roles, Grief and Loss, Healthy Relationships, Intimacy Needs, Introduction to Self-Help, Ongoing Care Needs, Spirituality, Stages of Change, and Thought Restructuring. Trained group facilitators enhanced emotional support, provided guidance about communication approaches, and addressed denial, as well as the process of grief and loss associated with the diagnosis of MCI in a loved one.

#### MSS Compensation Training

We provided each couple with MSS training 45 to 60 min daily with initial and ending adherence sessions. The curriculum is described briefly here.

##### Learning Phases

We utilized 3 training stages from learning theory [42]: (1) an acquisition phase in which use of the MSS is learned, (2) an application phase in which a participant is taught to apply MSS use to his/her daily life, and (3) an adaptation phase in which a participant practices incorporating the MSS into his/her daily life to make its use habitual and allow users to benefit from spared priming abilities in MCI [43].

##### Intervention Plan/Questions

This set of questions used in each training session was constructed to help the participants learn each training phase. These questions cover the topics to be learned in each phase of training. Participants progressed to the next training phase after demonstrating 100% accuracy on the intervention plan/questions in a stage for 2 consecutive days.

##### Homework

In addition to asking the intervention plan/questions, homework was given at the end of session to focus on the practice of an MSS skill.

##### Importance of the Care Partner

We were aware that even in cognitively intact people, 10 hours of direct training may be insufficient for the acquisition of a new procedural learning skill [44]. As such, we included a care partner in the training to help with cuing and practice outside of the therapy sessions.

##### Adherence

MSS adherence (ie, how well an individual utilizes all sections of their MSS calendar system) was defined as a score of 7 or greater on the adherence assessment. The adherence assessment was given on 5 occasions: on the first day of the intervention, the last day of the intervention, and 6, 12, and 18 months post HABIT. The evaluator examined MSS compliance for 2 days that are randomly selected from the prior week. Random days are selected to offset the possibility of a participant “preparing” the calendar for the evaluator’s visit.

### Longitudinal Outcomes and Booster Sessions

This study was originally proposed to study 6-month outcomes. However, our funding agency’s reviewers recognized that in typical neurodegenerative conditions, outcomes may diverge more clearly over time. Thus, Patient Centered Outcomes Research Institute required us to use the longitudinal time points tenable with the 5-year funding period. This was ultimately 18 months of follow-up. A robust literature suggests that “booster” sessions are helpful for behavioral interventions to have long-term impact [45]. We therefore had participants return at 6 and 12 months post intervention for a 1-day booster session where they first completed all follow-up measures and then received 1-hour sessions of the 4 intervention components of their particular study arm.

### Outcomes

Table 1 lists the measurement domains and measures in each domain. The table is organized according to which member of the dyad completed the measure. As previously determined in aim 1 of the study, patients’ QoL was our primary outcome.

**Table 1:** Treatment efficacy measures proposed to the patient and partner advisory group.

Target	Cognition	Physical function	Functional status	Mood	Quality of life	Self-efficacy	Care partner burden
Participant	Cogstate	SPPE^a^		CES-D^b^, AIF^c^	QoL-AD^d^	SE^e^ in MCI^f,g^	
Care partner		SPPE	ECog^h^, FAQ^i^	CES-D, AIF	QoL-AD	Caregiver SE	CB^j^

^a^SPPE: Short Physical Performance Examination.

^b^CES-D: Center for Epidemiological Studies Depression Scale.

^c^AIF: Anxiety Inventory Form.

^d^QoL-AD: Quality of Life-Alzheimer’s Disease.

^e^SE: self-efficacy.

^f^Modified from chronic disease Self-Efficacy Scales.

^g^MCI: mild cognitive impairment.

^h^ECog: Everyday Cognition.

^i^FAQ: Functional Assessment Questionnaire.

^j^CB: care partner burden.

#### Cognition

We assessed the patients’ cognitive function using Cogstate [46], a computerized measure of cognition. We used the Cogstate battery specifically designed for preclinical Alzheimer and MCI populations. This includes measures of simple and choice reaction time (the detection and identification tests, respectively), a test of visual memory (the One-Card Test) and a measure of working memory (One Back Test). This was completed at baseline and at 12-month follow-up.

#### Physical Function

We administered a timed 400-m walk and the Short Physical Performance Examination [47], which includes a timed 4-m walk, standing side by side, semitandem and full-tandem stance, and a timed arms-folded rise from seated to standing 4 times. This was completed at baseline, intervention completion, and 12-month follow-up.

#### Functional Status

Activities of daily living (ADL) functional status ratings based on informant assessment were obtained at baseline, intervention completion, and 6 months, 12 months, and 18 months post intervention. The Everyday Cognition (ECog) [48] was used to assess impairments in instrumental ADL. The ECog is an informant-based measure that assesses a participant’s ability to perform everyday tasks in the following areas: memory, language, visuospatial abilities, planning, organization, and divided attention. It was constructed specifically to be sensitive to changes in MCI. Factor analysis supports a 7-factor structure, including one global factor and 6 domain-specific factors. The global factor is strongly correlated with CDR score, MMSE, and clinical diagnosis. In addition to the global factor, the everyday memory factor differentiates MCI from normal cognition, and the everyday language factor differentiates MCI from dementia. Test-retest reliability over an average of 29 days is good (*r*=.82) [48]. The ECog was modified with its author’s support to assess the participant’s existing functional ability at each time point rather than the original wording comparing functioning with 10 years before to better gauge change from baseline to follow-up. Additionally, in anticipation of future long-term follow-up, ADL and instrumental ADL were further assessed with the Functional Assessment Questionnaire (FAQ) at baseline, intervention completion, and 6, 12, and 18 months post intervention. The FAQ is the standard functional measure required for use throughout the Alzheimer’s Disease Research Center’s network and was developed for use with dementia patients but has also shown to discriminate between normal controls and those with MCI [49]. In this regard, we surmised the FAQ would prove to be more useful in measuring advanced ADL impairments than the ECog in longitudinal follow-up.

#### Mood

At all assessment points, both the patient with MCI and the care partner completed the Center for Epidemiological Studies Depression Scale (CES-D). In addition, both the participant with MCI and the care partner completed the Anxiety Inventory Form, a 10-item rating scale modified from the State-Trait Anxiety Inventory [50] by the Resources for Enhancing Alzheimer’s Caregiver Health project [51].

#### Quality of Life

Both the participant and care partner completed the QoL-ADL [52]. The QoL-ADL is a 13-item measure developed for individuals with dementia that has been utilized in MCI and with care partners. Participants and care partners were asked to rate their relationships, concerns about finances, physical condition, mood, energy level, memory, aspects of daily functioning, and overall life quality on a 4-point scale.

#### Participant Self-Efficacy

The MCI participants completed a measure of self-efficacy at all assessment points using modified, selected items from the chronic disease Self-Efficacy Scales [53]. The entire 3-item Do Chores Scale, 2-item Social/Recreational Activities Scale, and 4 items of the 5-item Manage Disease in General Scale were utilized based on their relevance to MCI. Original scales have reported internal consistency reliability of *r≥*.82 and test-retest reliability of *r≥*.84 [53]. The language from the original scales was modified to be specific to those with MCI (ie, “your memory/cognitive difficulty” rather than more general references to “your health condition”). The result is the 9-item Self-Efficacy in Mild Cognitive Impairment Scale.

#### Care Partner Self-Efficacy

Care partners completed the caregiving competence and mastery components of the Pearlin [54] at all assessment points. The measures reflect their titles and range from 4 to 6 items.

Care partners completed the short form of the Caregiver Burden Inventory [55] at all assessment points. This measure is an assessment of degree of stress experienced by family caregivers. It includes 12 questions concerning the effect of the participant’s disability on care partners’ lives. It is scored as a composite measure, combining several aspects of caregivers’ reactions.

### Ancillary Data

#### Clinical Dementia Rating

Patients were given a CDR [56], which is a structured interview designed to stage dementia. In the CDR, the patient is rated on 6 dimensions: Memory, Orientation, Judgment and Problem Solving, Community Affairs, Home and Hobbies, and Personal Care; they are then assigned a global score, which is generated via an established algorithm. A global score of ≤0.5 was required for enrollment into the study. This measurement was updated at the 18-month visit to stage the patient’s cognitive impairment at study completion.

#### Global Cognitive Function

The Dementia Rating Scale, 2nd edition [57], was administered to help determine the overall level of cognitive functioning at baseline. This was also given at the 12-month follow-up point to characterize global cognitive functioning.

The MMSE [58] is a widely used screening measure of cognitive impairment. The MMSE was given to care partners at the eligibility session to determine whether global cognitive functioning is intact. A score of ˃24 was required for enrollment in the study.

#### Participant and Care Partner Self-Compassion

The participant with MCI and the care partner completed a measure of self-compassion at all assessment points using the 12-item short form of the Self-Compassion Scale [59]. This form has high internal consistency reliability of *r*=.87 and a very high correlation *r*=.97 with the full 26-item Self-Compassion Scale [60].

#### Participant and Care Partner Gratitude

The participant with MCI and the care partner completed the 6-item Gratitude Questionnaire Scale [61] at all assessment points. This Likert scale also has high internal consistency reliability of *r*=.82 and a high fit index (.95) for a single-factor model and good concurrent validity [61]. Table 2 lists the timing of the efficacy and ancillary measures used in the study.

**Table 2:** Timing of assessment measures.

Measure	Eligibility	Baseline	Intervention completion	6-month booster	12-month booster	18-month post (mail out)
DRS-2^a^		X			X	
MMSE^b^	X					
CDR^c^	X					By phone
Cogstate		X			X	
SPPE^d^		X	X		X	
Calendar adherence		X	X	X	X	By mail
Participant CES-D^e^		X	X	X	X	By mail
Participant QoL^f^		X	X	X	X	By mail
Participant SE^g^		X	X	X	X	By mail
Participant AIF^h^		X	X	X	X	By mail
Participant SC^i^		X	X	X	X	By mail
Participant G^j^		X	X	X	X	By mail
FAQ^k^		X	X	X	X	By mail
CB^l^		X	X	X	X	By mail
ECog^m^		X	X	X	X	By mail
Partner CES-D		X	X	X	X	By mail
Partner QoL		X	X	X	X	By mail
Partner SE		X	X	X	X	By mail
Partner AIF		X	X	X	X	By mail
Partner SC		X	X	X	X	By mail
Partner G		X	X	X	X	By mail
Activity log				X	X	By mail

^a^DRS-2: Dementia Rating Scale, 2nd edition.

^b^MMSE: Mini Mental State Exam.

^c^CDR: Clinical Dementia Rating.

^d^SPPE: Short Physical Performance Examination.

^e^CES-D: Center for Epidemiological Studies Depression Scale.

^f^QoL: quality of life.

^g^SE: self-efficacy.

^h^AIF: Anxiety Inventory Form.

^i^SC: self-compassion.

^j^G: gratitude.

^k^FAQ: Functional Assessment Questionnaire.

^l^CB: care partner burden.

^m^ECog: Everyday Cognition.

### Data Management

The study utilized Web-based electronic data capture in REDCap software [62] using forms previously created for use in our earlier study (Grant R01NR012419). The REDCap application uses PHP + JavaScript programming languages and a MySQL database engine. The forms were securely accessible at each site from computers or mobile devices with a Web browser. The data forms and data files are stored on a server hosted by the Mayo Clinic Center for Clinical and Translational Science (Grant UL1 RR024150).

### Data Analysis

#### Aim 1

Data analysis for aim 1 is complete and reported elsewhere.

#### Aim 2

To determine the degree to which the different components of the HABIT program contribute to improvements in each of the targeted outcomes, we will utilize linear mixed effect models approach that accounts for the randomized complete block design that was employed in the assignment of treatment combinations to study sessions within each site *.* In these models, we will include indicators for the different treatment effects, as well as indicators for potential confounding features such as age, sex, study site, and study session. Using these analyses, we will test for improvements from baseline over follow-up by testing for the significance of interactions between the variables representing study components and those reflecting follow-up period. These tests are similar to paired *t* tests but are more flexible. For example, they allow simultaneous analysis of data from more than 2 time points, and they enable the inclusion of covariates that may differ from baseline to follow-up.

We will examine the significance of the different treatment effects both with and without adjustment for potential confounders such as gender, age, and baseline scores on the Dementia Rating Scale. We will also explore potential heterogeneity of treatment effects by assessing interactions between treatments and gender, age, and baseline Dementia Rating Scale score of the patient and relationship of the partner (spouse vs other).

#### Aim 3

We aim to illustrate the efficiency of our novel design and statistical analysis methods for the evaluation of multicomponent interventions. The need for multicomponent studies is so critical that the Food and Drug Administration has gone so far to issue a plan for how to ensure combination (multicomponent) trials meet its standards [63]. Typical clinical trials often contrast one treatment to another (often a placebo). In the case where multiple treatments are studied, it is possible to employ fractional factorial designs [64]. In these designs, subsets of experimental combinations are carefully selected to enable the estimation of treatment effects of interest. As these designs compare an experimental condition with no active treatments to experimental condition with only one or two treatments, we opted to pursue a different experimental approach. In this approach, we formed 5 distinct treatment group combinations by removing a single component of the 5 possible components comprising the HABIT program. As part of this proposed effort, we will assess the efficiency of this study design in comparison with 2-arm studies (ie, treatment vs control) and fractional factorial designs. In our application of this study design, we randomly assigned one of these five treatment combinations to study session, stratified by study location in such a way that imbalance is minimized. Our existing study design meets the following constraints: (1) each study site (Arizona, Florida, Minnesota, and the University of Washington) administered each candidate treatment combination once and (2) each treatment combination was given 4+ times for a total of at least 20 sessions of treatment.

### Power Analysis

In aim 2, we will conduct a randomized trial to assess the ability of the different components of the HABIT program to effect change in patient QoL. As outlined above, analyses will be based on data gathered from approximately 300 individuals with amnestic MCI participating in one out of a total of 20 sessions. Randomized complete block study design was employed to assign treatment combinations within study sites. We used approaches developed for this design to estimate the power to conclude that a specific HABIT component provided benefit on the primary outcome. Data from our existing clinical sample and a matched, nonrandomized, untreated control group (collected for a different project by author JF) provided initial estimates for this simulation. We computed the expected variance of the estimated treatment effect for one HABIT component by extracting the appropriate value from the variance-covariance matrix derived from the design matrix corresponding to the allocation of treatment groups within the study. Using this, we estimated the magnitude of the effect size (difference score divided by its standard deviation) that is detectable with 80% power using a 2-sided .05 level test. The results of this effort suggest that we have 80% power to conclude that a treatment component is efficacious if it is associated with an improvement of 0.53 standard deviation units (*d*=0.53) while accounting for effects due to the other treatment components, study sites, and sessions within sites. We have observed differences larger than this in previous studies. For instance, we observed that training in the MSS improves ECog scores by nearly 0.9 standard deviations at first follow-up in a previous study [18]. Therefore, as we undertake the planned data analysis at study end, we will have sufficient power to detect the meaningful changes that we expect to observe.

## Results

**Figure 1:**
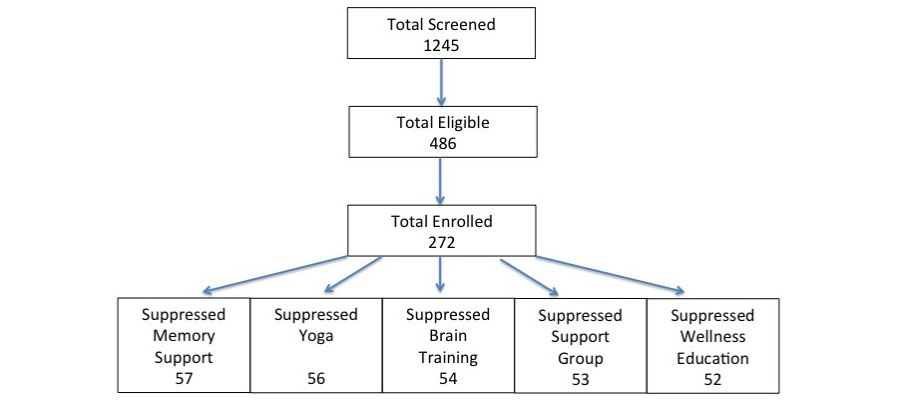
Recruitment and enrollment.

Enrollment for the trial began in September 2014 and was completed in August 2016. Figure 1 depicts our enrollment success. We screened all patients seen by our general clinical diagnostic neuropsychology services as potential candidates for the HABIT program (N=1245). Most of our patients are not candidates for HABIT because they did not meet MCI due to AD criteria (eg, they were cognitively normal after evaluation or had progressed to dementia or had cognitive impairment due to another known neurologic disorder such as epilepsy). A less common reason a patient would not be a candidate for HABIT was the absence of a study partner. Eventually we determined that 486 of our patients seen during the study enrollment period were eligible for the trial. Of those, 272 consented to participate in study. Past research suggests the primary reasons for nonenrollment of potential participants were time and distance involved, which are required to receive the intervention [35]. Thus, in our enrollment window, we were able to enroll a little over 90% of our targeted 300 participants. However, this required the conduct of extra HABIT sessions at each Mayo site. Ultimately, Mayo Minnesota, Mayo Florida, and Mayo Arizona conducted 6 HABIT sessions each. The University of Washington, with its later start, conducted 5 sessions. Table 3 lists site by arm enrollments. Demographics for the overall enrolled sample are listed in Table 4. Longitudinal follow-up to our key time points of 6, 12, and 18 months is partially completed and is now ongoing. Follow-up time visits will continue through February 2018.

**Table 3:** Site by arm enrollment; per arm.

Suppressed	Yoga	Group	Education	Brain fitness	Memory support system	Total enrolled
Arizona	14	15	17	21 (11+10)	18	85
Florida	13 (5+8)	13	8	9	10	53
Minnesota	22	17	19 (9+10)	12	19	89
University of Washington	7	8	8	12	10	45
Total	56	53	52	54	57	272

**Table 4:** Sample demographics.

Variable	Participants (N=272)	Partners (N=272)
Age, mean (SD^a^)	75.2 (7.6)	70.3 (10.1)
Education, mean (SD)	16.1 (2.8)	16.1 (2.7)
Female, n (%)	111 (40.8)	187 (68.7)
Nonwhite, n (%)	11 (4.0)	16 (5.9)
Cohabiting with study partner, n (%)	239 (87.9)	
Spouse of participant, n (%)		226 (83.1)
Median income, in US dollars	$75,000-99,000	$75,000-99,000
Taking anti-depressant, n (%)	106 (39.0)	60 (22.1)
Taking memory medication, n (%)	103 (37.9)	
Mini Mental State Exam, mean (SD)		28.9 (1.3)
Dementia Rating Scale, mean (SD)	128.8 (11.2)	

^a^SD: standard deviation.

## Discussion

This paper describes the protocol for the study “Comparative Effectiveness of Behavioral Interventions to Prevent or Delay Dementia” (ClinicalTrials.gov Identifier: NCT0226575). Included is a description of the rationale, design, power analysis, and analysis plan. Moreover, because enrollment is complete and we are in follow-up phases of the study, we have included enrollment data from the trial.

### Strengths

The study has several strengths. First, this is comparative effectiveness research, permitting comparison of different behavioral interventions. Second, the primary outcome was selected by prior patients. All participants received some form of treatment. The patient-centered comparative effectiveness design features have supported high retention of participants. Finally, the participants are well characterized.

### Limitations

There are also several key limitations of this study. This was not a double-blinded trial, so the investigator was aware of the intervention component that was missing and could conceivably bias outcomes. Informed consent also resulted in participants’ awareness that they are missing one component (eg, education), possibly impacting their expectations or leading them to identify and initiate that component on their own (eg, exercise). In an attempt to measure this possibility, we inquired at follow-up visits about other activities individuals engaged in outside of HABIT recommendations.

In addition, a weakness of our chosen comparative effectiveness design is that it does not permit comparison with no treatment. Still, our rather unique design of suppressing one of five treatments should allow us to examine the contribution of each component to the primary and secondary outcomes. Also, this grant period runs for only 3 years, limiting the number of sessions and length of the follow-up we can achieve for these participants.

This intervention targeted participants with amnestic MCI. This is because the intervention targeted memory impairments consistent with likely underlying AD pathology. This means we did not address other cognitive deficits such as language dysfunction. As such, these results may not be generalizable to individuals with nonamnestic MCI subtypes. These populations could certainly be targets for future research. Moreover, our cohort not well representative of the general population with MCI. Our cohort has high education attainment and high socioeconomic status. The treatment is intense, requiring 4 hours of participation per day, Monday through Friday for 2 weeks. This level of intensity may have served to further limit patient and partners’ ability to participate.

Finally, we do not believe this intervention will have disease-modifying effects and therefore have no mechanism for assessing whether it did. The goal of the study is to assess the impact on QoL, self-efficacy, functional status, and other mood-related variables for individuals with amnestic MCI (and their partners) despite the probable progression of their disease pathology. We anticipate publication of our findings within the next couple of years.

## Abbreviations

AD: Alzheimer disease
CDR: clinical dementia rating
CES-D: Center for Epidemiological Studies Depression Scale
DVD: digital video disc
ECog: Everyday Cognition
FAQ: Functional Assessment Questionnaire
HABIT: Healthy Actions to Benefit Independence and Thinking
MCI: mild cognitive impairment
MMSE: Mini Mental State Exam
MSS: Memory Support System
QoL: Quality of Life
